# Hypercoagulable states in young adults with ischemic stroke in a Stroke Belt state: a retrospective study

**DOI:** 10.3389/fneur.2024.1393999

**Published:** 2025-01-06

**Authors:** David Lee Gordon, Sarah R. Durica

**Affiliations:** Department of Neurology, University of Oklahoma Health Sciences Center, Oklahoma City, OK, United States

**Keywords:** arterial thrombosis, hypercoagulable state, ischemic stroke, Stroke Belt, thrombophilia

## Abstract

We theorize that the southeastern United States has a higher stroke mortality rate and higher recurrent ischemic stroke rate than the rest of the United States due to (1) an increased prevalence of hypercoagulable states among young adults in the region, (2) failure to diagnose hypercoagulable states as the cause of ischemic stroke in young adults, and (3) underutilization of anticoagulation for ischemic stroke secondary prevention in young adults with hypercoagulable states. In an attempt to investigate this hypothesis, we conducted a retrospective chart review of 311 inpatients with first-ever ischemic stroke from age 18 to 55 years at an Oklahoma academic medical center from 1 July 2011 to 30 April 2017. Using Chi-squared test, we compared the stroke etiologic diagnosis of the attending neurologist at discharge—when hypercoagulable profile results were rarely available—to the diagnosis of a vascular neurologist postdischarge who had access to all available etiologic test results. The inpatient neurologists identified the stroke etiology as hypercoagulable state in 79 patients (25.57%) and undetermined etiology in 105 (33.98%). With the benefit of final hypercoagulable profile results, the postdischarge vascular neurologist identified the stroke etiology as hypercoagulable state in 167 (54.22%, 95% CI 48.64–59.70%, *p*-value <2.2e-16) and undetermined etiology in 28 (9.09%, 95% CI 6.36–12.83%, *p*-value 1.03e-14). There was no significant change in the proportion of ischemic strokes classified under all other stroke etiologies. We conclude that hypercoagulable states are a common and underrecognized cause of ischemic stroke in young adults in the U.S. Stroke Belt.

## Introduction

The Stroke Belt is an area of the southeastern United States with the highest age-adjusted stroke mortality rate among adults (1), highest incidence of recurrent ischemic stroke among adults (2), highest stroke mortality rate among children (3), and highest age-adjusted heart-failure mortality rate among adults (4). Investigators have concluded that traditional (Framingham) vascular risk factors and low socioeconomic status explain only 32% of the increased stroke prevalence in the Stroke Belt (5); that those living in the Stroke Belt during their teen years have the highest lifetime stroke risk (6); and that “stroke risk factors that are applicable to both children and adults should be considered in attempts to explain” the Stroke Belt phenomenon (3).

The Stroke Belt usually includes ten states: Alabama, Arkansas, Georgia, Kentucky, Louisiana, Mississippi, North Carolina, South Carolina, Tennessee, and Virginia. While usually not considered a Stroke-Belt State, Oklahoma lies at the northwest corner of the Stroke Belt and historically has had among the highest mortality rates in the United States for stroke, coronary heart disease, and overall cardiovascular disease—higher, in fact, than many of the Stroke-Belt states. In 2007, for example, among the 50 states, District of Columbia, and Puerto Rico, Oklahoma ranked 48th (fifth worst) for stroke mortality, 51^st^ (second worst) in coronary heart disease mortality, and 50th (third worst) for overall cardiovascular disease mortality per the American Heart Association (AHA)/American Stroke Association (ASA) data (7).

We theorize that the southeastern United States has a higher stroke mortality rate and higher recurrent ischemic stroke rate than the rest of the United States due to (1) an increased prevalence of hypercoagulable states among young adults in the region, (2) failure to diagnose hypercoagulable states as the cause of ischemic stroke in young adults, and (3) underutilization of anticoagulation for ischemic stroke secondary prevention in young adults with hypercoagulable states.

Our study aims to determine the prevalence of hypercoagulable states as a cause of ischemic stroke in young adults at a major academic center in the southeastern United States and provide a potential explanation for the lack of recognition for hypercoagulable states among this patient population.

## Study methods

Since 2007, the stroke service at the University of Oklahoma has performed comprehensive hypercoagulable profiles on nearly all young adults with ischemic stroke in whom there is no clear arterial or cardiac etiology. This includes patients with small-artery occlusion since young adults rarely have hypertension or diabetes mellitus long enough to develop significant arterial disease. The contents of the hypercoagulable profile expanded after the first few years to include factor XI and methylenetetrahydrofolate reductase (MTHFR) variants. Table 1 depicts the latest version of our hypercoagulable profile contents with corresponding values considered to be positive. Final results of most of these laboratory tests take several days to weeks and, thus, are not available at the time of patient discharge from the hospital when the attending stroke neurologist must determine the likely etiology and prescribe antithrombotic therapy for secondary stroke prevention.

**Table 1:** University of Oklahoma comprehensive hypercoagulable profile.

Hypercoagulable profile components	Value considered positive
Excessive clotting factors	
Fibrinogen	Per laboratory range and if also elevated after 8 weeks
Factor VII	Per laboratory range
Factor VIII	Per laboratory range and if also elevated after 8 weeks
Factor XI	Per laboratory range
Deficient natural anticoagulants	
Antithrombin	Per laboratory range
Protein C	Per laboratory range
Protein S (total and free)	Per laboratory ranges
Genetic variants	
Activated protein C resistance	Per laboratory range
Factor V Leiden	Heterozygote or homozygote variant
Prothrombin G20210A	Heterozygote or homozygote variant
Methylenetetrahydrofolate reductase 665C>T	Heterozygote or homozygote variant
Methylenetetrahydrofolate reductase 1286A>C	Heterozygote or homozygote variant
Antiphospholipid antibodies	
Lupus anticoagulant	Positive confirmatory tests
Anticardiolipin antibodies IgG, IgM	Per laboratory range
Anti-beta-2-glycoprotein I antibodies IgG, IgM	Per laboratory range
Antiphosphatidylserine antibodies IgG, IgM	Per laboratory range
Other	
Lipoprotein (a)	Greater than 30 mg/dL
Sickle cell screen	Per laboratory conclusion
C-reactive protein	Per laboratory range
Complete blood count	
Hemoglobin	Per laboratory range
Platelet count	Per laboratory range

We conducted a retrospective chart review of all patients admitted to the University of Oklahoma Medical Center with ICD-9 codes 433, 434, 436 or ICD-10 code I63 from 1 July 2011 to 30 April 2017 and identified 311 with first-ever ischemic stroke ages 18 to 55 years. From a thorough manual review of each record, we identified the ischemic stroke etiologic diagnosis of the attending neurologist at discharge.

Using Chi-squared test, we compared the stroke etiologic diagnosis of the attending neurologist at discharge—when hypercoagulable profile results were rarely available—to the diagnosis of a vascular neurologist postdischarge who had access to all available etiologic test results. All neurologists used a modified Trial of Org 10,172 in Acute Stroke Treatment (TOAST) classification with 7 categories: large-artery atherosclerosis, cardioembolism, small-artery occlusion, hypercoagulable state, nonatherosclerotic vasculopathies, hypoperfusion, and stroke of undetermined etiology. The TOAST investigators designed the original TOAST classification for use in multicenter randomized clinical trials of acute ischemic stroke, so emphasized the three most common ischemic stroke etiologies, large-artery atherosclerosis, cardioembolism, and small-vessel occlusion, and included all other possible ischemic stroke etiologies under the umbrella “stroke of other determined etiology” (8). In the modified TOAST classification used in this study, we divided “stroke of other determined etiology” into three specific categories: hypercoagulable state, nonatherosclerotic vasculopathy, and hypoperfusion (Table 2). In the class “stroke of undetermined etiology,” we included strokes with two or more identified causes and strokes with either a negative or incomplete evaluation.

**Table 2:** TOAST classification of ischemic stroke subtypes.

Original TOAST classification	Modified TOAST classification
Large-artery atherosclerosis (embolus/thrombosis) Cardioembolism Small-vessel occlusion (lacune) Stroke of other determined etiology Stroke of undetermined etiology Two or more causes identified Negative evaluation Incomplete evaluation	Large-artery atherosclerosis (embolus/thrombosis) Cardioembolism Small-artery occlusion (lacune) Hypercoagulable state (thrombophilia) Nonatherosclerotic vasculopathy Hypoperfusion (hypotension, watershed, borderzone) Stroke of undetermined etiology Two or more causes identified Negative evaluation Incomplete evaluation

The postdischarge neurologist determined a patient’s hypercoagulable profile results were the likely cause of ischemic stroke based on the criteria in Table 3, summarized as follows:

- Infarction in large-artery or lenticulostriate or thalamoperforator territory with no potential arterial, cardiac, or aortic source of thromboembolism

**Table 3:** Criteria for hypercoagulable state as ischemic stroke etiology in young adults (see text for clarifications regarding patent foramen ovale, fibrinogen, and factor VIII).

Infarction in large-artery or lenticulostriate or thalamoperforator territory with no potential arterial, cardiac, or aortic source of thromboembolism No potential arterial source—corresponding large artery stenosis <50% by catheter angiography or < 70% by CT angiography, MR angiography, or carotid duplex ultrasound; no evidence of cervicocephalic arterial dissection, moyamoya disease, or arteritis No potential cardiac source—echocardiogram shows ejection fraction >35%, absence of left-ventricular wall-motion abnormalities, normal left atrial size, lack of intracardiac thrombi or tumor, and lack of aortic or mitral valvular vegetations; electrocardiogram shows no evidence of atrial fibrillation, atrial flutter, or sick sinus syndrome No potential aortoembolic source—transesophageal echocardiogram shows aortic arch plaque <4 mm thickness OR Infarction in anterior choroidal or pontine artery territory (small-artery territories which are rarely due to emboli and rarely due to small-artery disease in young patients) AND Any combination of the following hypercoagulable profile results: Fibrinogen level elevated both at time of stroke and 8–12 weeks later Factor VII level elevated Factor VIII level elevated both at time of stroke and 8–12 weeks later Factor XI level elevated Anthithrombin level decreased Protein C level decreased Protein S free level decreased Activated protein C resistance decreased Factor V Leiden variant heterozygote or homozygote Prothrombin G20210A variant heterozygote or homozygote Methylenetetrahydrofolate reductase (MTHFR) 665C>T variant heterozygote or homozygote Methylenetetrahydrofolate reductase (MTHFR) 1286A>C variant heterozygote or homozygote Lupus anticoagulant positive Anticardiolipin antibodies IgG or IgM levels elevated Anti-beta-2-glycoprotein I antibodies IgG or IgM levels elevated Antiphosphatidylserine antibodies IgG or IgM levels elevated Lipoprotein (a) level greater than 30 mg/dL

OR

- Infarction in anterior choroidal or pontine artery territory (small-artery territories which are rarely due to emboli and rarely due to small-artery disease in young patients)

AND

- Positive hypercoagulable results.

Note that we defined significant large-artery stenosis based on the criteria of the Carotid Revascularization Endarterectomy vs. Stenting Trial (CREST), i.e., 50% stenosis by catheter angiography or 70% stenosis by CT angiography, MR angiography, or carotid duplex ultrasound stenosis (9).

Also note that we defined significant ejection fraction as ≤35% based on consensus guidelines stating that cardioembolism is most likely with this degree of left ventricular dysfunction (10).

We did not consider patent foramen ovale (PFO) as a cause of ischemic stroke because, in our opinion, PFOs are either incidental findings or a secondary finding in patients with hypercoagulable state (i.e., providing an additional mechanism for thrombophilia to become symptomatic). Approximately 25% of all people have a patent foramen ovale (11). The likelihood that a PFO is coincidental is higher in older patients who commonly have vascular risk factors for many years. For this reason, studies that show no difference between anticoagulation and antiplatelet therapy in ischemic stroke patients greater than 55 years of age with PFO have not influenced our diagnostic or management decision making (12). Furthermore, (1) PFOs are clearly a conduit for venous—not arterial—thromboembolism, (2) clotting factors—not platelets—cause venous thromboembolism, and (3) the treatment of venous thromboembolism is anticoagulation—not antiplatelet therapy. Yet three recent studies that reported benefit of PFO closure over optimal medical management in young adults with ischemic stroke either compared only antiplatelet agents vs. PFO closure (13) or compared both antiplatelet agents and anticoagulation together vs. PFO closure and had insufficient number of anticoagulation patients to determine possible benefit of anticoagulation over PFO closure (14, 15).

Isolated elevations of fibrinogen and factor VIII levels are usually acute-phase responses that occur as a result of thrombosis rather than a primary cause of thrombosis. Obtaining these tests in the acute phase is still valuable as a marker of active thrombosis. The likelihood of acute-phase response is higher when fibrinogen level, factor VIII level, and C-reactive protein are all elevated concurrently. Persistent elevations of fibrinogen or factor VIII, however (for example, eight to twelve weeks after stroke occurrence), suggest the elevations of these coagulation factors are causative rather than reactive.

## Study results

The mean age of the subjects was 46.1 (± 10.7). Table 4 contains the subjects’ pertinent demographic data, including the number of subjects with traditional “Framingham” vascular risk factors. We included these data to demonstrate that many patients with hypercoagulability also have traditional vascular risk factors, but we do not believe that these risk factors contribute to the etiology of ischemic stroke in patients with hypercoagulable states.

**Table 4:** Subjects’ demographic data.

Category	Results (Total *N* = 311)
Age, mean (SD)	46.1 (± 10.7)
Age, median (Q1, Q3)	48.0 (39.0, 55.0)
Gender, female	141 (45.3%)
Hypertension	142 (45.7%)
Diabetes mellitus	75 (24.1%)
Hyperlipidemia	85 (27.3%)
Coronary artery disease	36 (11.6%)
Cigarette smoking	124 (39.9%)

SD, standard deviation. Q1 = first quartile data point (25% of values are lower). Q3 = third quartile data point (75% of values are lower).

At discharge, inpatient neurologists classified an ischemic stroke etiology in 309 subjects because two patients died soon after admission before undergoing etiologic evaluation. The postdischarge vascular neurologist classified an ischemic stroke etiology in 308 subjects due to a missing (paper) medical record of one subject.

**Figure 1:**
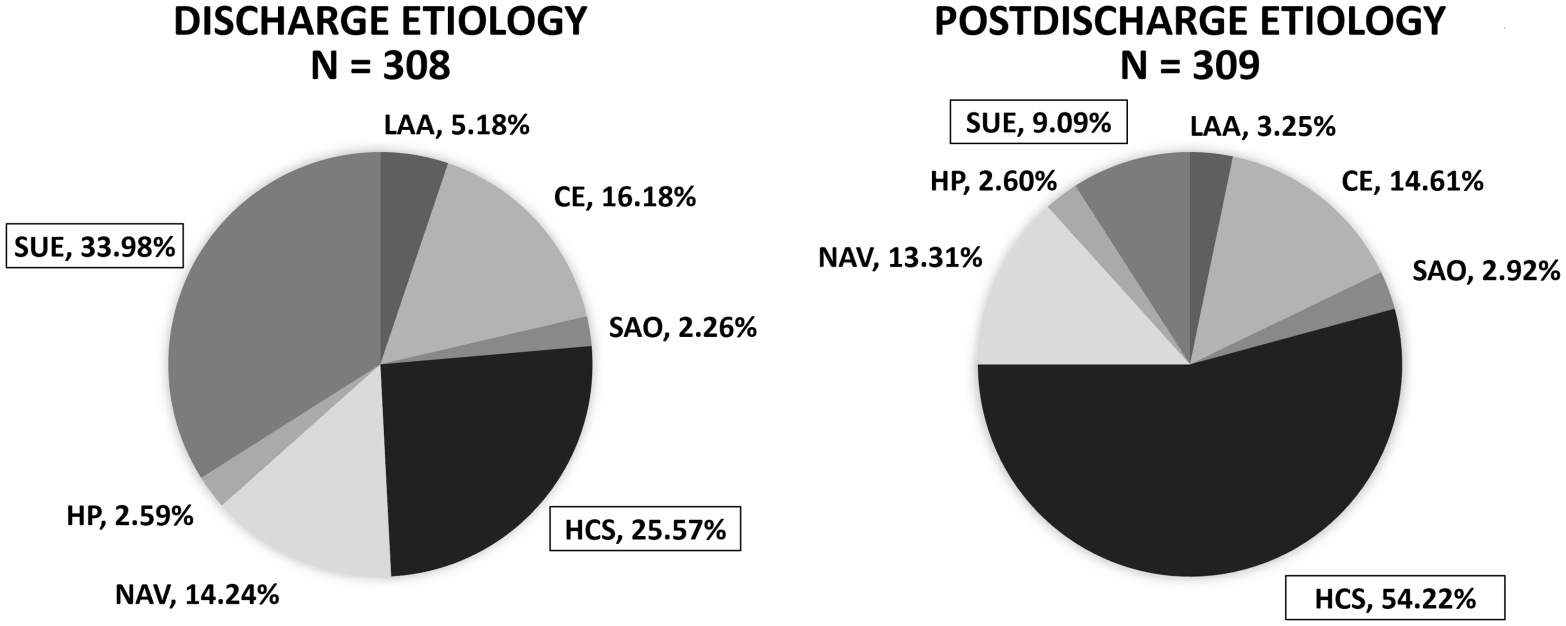
Influence of hypercoagulable profile results on perceived ischemic stroke etiology in young adults (discharge vs. postdischarge when hypercoagulable profile results are known). After knowledge of the hypercoagulable profile results, a postdischarge vascular neurologist determined significantly more patients had ischemic stroke due to hypercoagulable state (HCS) (54.22%, 95% confidence interval 48.64–59.70%, *p*-value <2.2e-16) and significantly less patients had stroke of undetermined etiology (SUE) (9.09%, 95% confidence interval 6.36–12.83%, *p*-value 1.03e-14). LAA, large-artery atherosclerosis; CE, cardioembolism; SAO, small-artery occlusion; HCS, hypercoagulable state; NAV, nonatherosclerotic vasculopathy; HP, hypoperfusion; SUE, stroke of undetermined etiology.

The inpatient neurologists identified the stroke etiology as hypercoagulable state in 79 patients (25.57%) and undetermined etiology in 105 patients (33.98%). With the benefit of final hypercoagulable profile results, the postdischarge vascular neurologist identified the stroke etiology as hypercoagulable state in 167 subjects (54.22%, 95% CI 48.64–59.70%, *p*-value <2.2e-16) and undetermined etiology in 28 subjects (9.09%, 95% CI 6.36–12.83%, *p*-value 1.03e-14); there was no significant change in the proportion of ischemic strokes classified under all other stroke etiologies (Table 5; Figure 1).

**Table 5:** Influence of hypercoagulable profile results on perceived ischemic stroke etiology in young adults (discharge vs. postdischarge when hypercoagulable profile results are known).

		LAA	CE	SAO	HCS	NAV	HP	SUE	Total
Discharge ischemic stroke etiology	Number	16	50	7	79	44	8	105	309
	Percent	5.18%	16.18%	2.26%	25.57%	14.24%	2.59%	33.98%	100%
	95% confidence interval	(3.21, 8.24%)	(12.49, 20.70%)	(1.10, 4.60%)	(21.02, 30.71%)	(10.78, 18.58%)	(1.32, 5.02%)	(28.93, 39.43%)	
Postdischarge ischemic stroke etiology	Number	10	45	9	167	41	8	28	308
	Percent	3.25%	14.61%	2.92%	54.22%	13.31%	2.60%	9.09%	100%
	95% confidence interval	(1.77, 5.87%)	(11.10, 18.99%)	(1.54, 5.46%)	(48.64, 59.70%)	(9.97, 17.56%)	(1.32, 5.04%)	(6.36, 12.83%)	

LAA, large-artery atherosclerosis; CE, cardioembolism; SAO, small-artery occlusion; HCS, hypercoagulable state; NAV, nonatherosclerotic vasculopathy; HP, hypoperfusion; SUE, stroke of undetermined etiology.

## Discussion

In our study, among young adults with ischemic stroke, a vascular neurologist with knowledge of the final hypercoagulable profile results diagnosed hypercoagulable state as the cause of stroke significantly more often and stroke of undetermined etiology significantly less often than neurologists who cared for the patient but were not aware of the final hypercoagulable profile results.

The AHA/ASA stroke guidelines are inconsistent regarding hypercoagulable states as a potential cause of ischemic stroke, the need to perform hypercoagulable profiles, and the treatment of patients with hypercoagulable states. The AHA/ASA Acute Ischemic Stroke guidelines of 2019 recommend NOT testing for inherited thrombophilia in patients with stroke, myocardial infarction, or peripheral thrombosis (16). The AHA/ASA Stroke and TIA Prevention guidelines of 2021 state that it is “reasonable to perform” tests for hypercoagulable states in patients with cryptogenic stroke, but that antiplatelet therapy is “reasonable” treatment for patients with these conditions (17). The AHA/ASA Stroke in Neonates and Children guidelines of 2019 recommend a thrombophilia evaluation for “every case” of non-neonatal childhood stroke and treatment with anticoagulants in these patients (18).

In fact, the role of hypercoagulable states in both venous and arterial thrombosis in general is controversial. Most major medical organizations advise against testing for thrombophilia in patients with venous thromboembolism (19) and the role of thrombophilia in arterial thrombosis is even more uncertain as evidenced by the variable AHA/ASA recommendations (20).

There are challenges in establishing hypercoagulable states as a cause of ischemic stroke. The most difficult challenge is the fact that individual abnormalities are often asymptomatic in isolation. The normal thrombotic mechanism has tremendous redundancy, presumably an evolutionary mechanism to increase the odds of survival. Symptomatic thrombosis is uncommon if a patient has only one hypercoagulable state and is more likely if a patient has two or more conditions contributing to thrombophilia—for example, two or more concurrent hypercoagulable states, one hypercoagulable state plus an atrial septal abnormality (PFO or interatrial septal aneurysm), or one hypercoagulable state plus persistent migraine aura (with concurrent prolonged oligemia and stasis of flow). The need for more than one abnormality to cause symptomatic thrombosis has been called the “multiple hit hypothesis.”

Adding to the uncertainty is that some hypercoagulable abnormalities may occur transiently. During infections or acute inflammatory states, for example, fibrinogen and factor VIII levels increase and free protein S levels decrease (due to increased production of C4b-binding protein with increase in bound protein S). While some hematologic or vascular experts argue that these transient changes are of no consequence, they may either cause or signal a transiently increased risk for thrombosis and explain why stroke and heart risk increases during infections (21). In addition, antiphospholipid antibodies may increase transiently during autoimmune flares—with resultant venous or arterial thrombosis at that time—but return to lower, nonpathologic levels several weeks later (22). The transient nature of these hypercoagulable states limits their detection in population-based studies and when thrombophilia testing is delayed for weeks after thrombosis occurrence and, thus, casts doubt on their perceived role in causing thrombosis.

Many small studies have suggested an increased risk of thrombosis in patients with multiple hypercoagulable hits. In a study of patients with symptomatic thrombosis and inherited thrombophilia, 10–30% had one defect and 75% had two defects (23). In a study of patients with symptomatic thrombosis and inherited thrombophilia, 50% had an additional acquired risk factor (24). In a study of patients with symptomatic thrombosis and protein C deficiency, 40% also had factor V Leiden variant; in the same study, 29% of patients with symptomatic thrombophilia and protein S deficiency also had factor V Leiden variant (25). In a study of patients with antiphospholipid (APL) syndrome, the risk of arterial thrombosis increased with concurrent presence of MTHFR 665C>T (= 677 T) variant (26). In a study of symptomatic thrombosis in children with ischemic stroke and cardiac disorders, 10.5% had more than one hypercoagulable state versus none in control subjects (27). In a study of patients younger than 55 years old with ischemic stroke and PFO compared to age-matched controls, the stroke-PFO patients tested significantly more often for factor V Leiden variant or prothrombin G20210A variant (28).

The need for two or more hits explains why inherited thrombophilia may be asymptomatic for many years, especially on the arterial side. Stasis is a second hit and, since stasis is more likely in the venous circulation, thrombophilia more commonly causes venous thrombosis. With a sufficient number of hits, however, thrombophilia can also cause arterial thrombosis. As cited in the paragraph above, patients with APL syndrome have increased risk of arterial thrombosis if they also have MTHFR mutation; interpreted another way, patients with MTHFR variant are at increased risk of arterial thrombosis vs. those without the variant if they suffer a second hit such as APL syndrome (26). In another example, investigators in a recent meta-analysis concluded that specific inherited thrombophilias (factor V Leiden variant, prothrombin G20210A variant, protein C deficiency, and protein S deficiency) are associated with an increased risk of arterial ischemic stroke in adults (20). Based on these, other studies, and our own experiences, we have concluded that the segregation of venous and arterial thrombophilia is an artificial one based on false premises.

Accepting the premises that hypercoagulable states are more common than previously appreciated and often symptomatic explains several otherwise mysterious conditions, including ischemic stroke in young adults, the United States Stroke Belt in adults and children, economy class syndrome (occurrence of deep-vein thrombosis in some patients after prolonged immobility on long airplane fights), MINOCA (Myocardial Infarction with NonObstructive Coronary Arteries found in approximately 10% of patients with myocardial infarction) (29), and myocardial infarction in young adults (significantly more likely to have normal coronary angiography, nonobstructive coronary artery disease, or single-vessel obstruction than older patients) (30).

It is important to note that having traditional ischemic stroke risk factors alone does not necessarily mean that a patient’s ischemic stroke is related to those risk factors. Risk factors are not etiologies and are unrelated to stroke etiology if there is no associated arterial or cardiac disease. In our study, many patients had traditional ischemic stroke risk factors such as hypertension (45%) and cigarette smoking (39.9%), but we considered these risk factors coincidental if they had normal arterial and cardiac diagnostic testing.

Our study was retrospective and, as such, subject to selection bias and incomplete or unavailable records. The retrospective data collection, however, is partially offset by our inclusion of all young adults with a coded ischemic stroke diagnosis during the study period as well as our institution’s routine practice of obtaining comprehensive hypercoagulable profiles in all young adults with ischemic stroke and normal arterial and cardiac evaluations. Additionally, given the retrospective nature of our study, we were unable to evaluate long-term outcomes of patients with hypercoagulable stroke etiology treated with anticoagulation. Our sample size was also small and restricted to patients evaluated at a single academic stroke center, which may limit generalizability of the results. Finally, our results depended on the interpretation of a single stroke neurologist. Future multicenter studies evaluating the prevalence of hypercoagulable states in young adults with stroke, as well as the efficacy of long-term anticoagulation in these patients, are necessary.

Our study results are consistent with our theory that hypercoagulable states are a common cause of ischemic stroke in young adults in Oklahoma, a state adjacent to the U.S. Stroke Belt with vascular mortality data comparable to states traditionally considered members of the Stroke Belt. Furthermore, the significant difference of opinion regarding ischemic stroke etiology before and after receiving results of a hypercoagulable profile suggests that failure to perform a hypercoagulable profile in these patients may result in lost opportunities to optimize secondary stroke prevention strategies and may at least partially account for the increased risk of stroke mortality and recurrence in the Stroke Belt.

## Data Availability

The raw data supporting the conclusions of this article will be made available by the authors, without undue reservation.
